# Integrated community-based HIV and sexual and reproductive health services for youth: a cluster-randomized trial

**DOI:** 10.1038/s41591-025-03762-z

**Published:** 2025-06-24

**Authors:** Rashida A. Ferrand, Ethel Dauya, Chido Dziva Chikwari, Tsitsi Bandason, Sarah Bernays, Constance Mackworth-Young, Aoife M. Doyle, Chris Grundy, Pitchaya Indravudh, Fern Terris-Prestholt, Constancia Vimbayi Mavodza, Owen Mugurungi, Tsitsi Apollo, Getrude Ncube, Leyla Larsson, Ona McCarthy, Victoria Simms, Mandikudza Tembo, Katharina Kranzer, Richard J. Hayes

**Affiliations:** 1 Clinical Research Department, London School of Hygiene & Tropical Medicine, London, UK; 2 The Health Research Unit Zimbabwe, Biomedical Research and Training Institute, Harare, Zimbabwe; 3 Department of Infectious Disease Epidemiology, London School of Hygiene & Tropical Medicine, London, UK; 4 Faculty of Medicine and Health, University of Sydney, Sydney, New South Wales Australia; 5 Department of Global Health and Development, London School of Hygiene & Tropical Medicine, London, UK; 6 Department of Public Health, Environments and Society, London School of Hygiene & Tropical Medicine, London, UK; 7 AIDS and TB Unit, Ministry of Health and Child Care, Harare, Zimbabwe; 8 Institute of Infectious Diseases and Tropical Medicine, LMU University Hospital, Munich, Germany; 9 Department of Population Health, London School of Hygiene & Tropical Medicine, London, UK

**Keywords:** Epidemiology, Developing world

## Abstract

Human immunodeficiency virus (HIV) viral suppression rates are disproportionately worse in youth compared to other age groups, and improving this will require addressing the whole HIV cascade, including HIV testing, linkage to care and support to maintain viral suppression. We conducted a cluster-randomized trial of community-based services incorporating HIV testing, treatment and adherence support integrated with sexual and reproductive health (SRH) services for youth (16–24 years) in Zimbabwe. Our hypothesis was that integrated services in community-based settings would increase demand and access. In total, 24 clusters (geographically demarcated areas) were randomized 1:1 to intervention or control (existing services). Primary outcome was virological suppression (defined as HIV viral load <1,000 copies per ml) among youth with HIV (YWH), ascertained through a population-level outcome survey of 17,682 youth (18–24 years). Secondary outcomes, corresponding to UNAIDS 90-90-90 targets, were the proportion of YWH who knew their HIV status, the proportion of YWH who knew their HIV status who were taking antiretroviral therapy (ART) and the proportion of YWH taking ART who achieved viral suppression (HIV viral load <1,000 copies per ml). There was no difference by arm in primary outcome (mean cluster prevalence—41.3% (intervention) versus 38.3% (control); risk ratio (RR)—1.07 (95% confidence interval (CI), 0.88–1.30)) or in proportion of YWH who were diagnosed. In the intervention arm, a lower proportion of diagnosed YWH were taking treatment (RR = 0.91 (95% CI, 0.83–0.99)), but a higher proportion of those on ART had viral suppression (RR = 1.18 (95% CI, 1.02–1.37)). The intervention did not impact the proportion of youth with undiagnosed HIV, which explains the lack of effect on the primary outcome. Among those taking treatment, the intervention improved viral suppression. Delivery of integrated HIV and SRH services was feasible and facilitated uptake by youth of essential services beyond HIV, addressing an important programmatic gap. Trial registration number: NCT03719521.

## Abstract (web summary)

Delivery of human immunodeficiency virus (HIV) testing, treatment and adherence support integrated into sexual and reproductive health services for youth in Zimbabwe did not improve population-level HIV viral suppression over standard care, largely due to a substantial proportion of youth with undiagnosed HIV—highlighting implementation challenges to achieve UNAIDS 90-90-90 targets.

## Main

The general global decline in new human immunodeficiency virus (HIV) infections has been much less marked in youth. In 2019, 30% of HIV infections in eastern and southern Africa occurred among women aged 15–24 years^1^. Compared to other age groups, youth living with HIV are less likely to be diagnosed, and those diagnosed have lower rates of HIV viral suppression once they start antiretroviral therapy (ART)^2^.

HIV testing is a prerequisite for accessing care or prevention services. Population-based surveys from sub-Saharan Africa, where two-thirds of the world’s population with HIV lives, show a substantial burden of undiagnosed HIV infection among youth. Only 52%, 48% and 45% of those aged 15–24 years in Zimbabwe, Malawi and Zambia, respectively, reported ever having an HIV test in population HIV impact assessments (PHIAs) conducted between 2015 and 2017^3–5^. In these countries, it was estimated that only 40–50% of 15–24-year-olds living with HIV were aware of their HIV status compared with 66–73% of those aged >24 years. Similarly, HIV viral load suppression among youth taking ART was significantly lower than among older people. Youth are therefore a priority group to achieve HIV control. Viral non-suppression is associated not only with morbidity but also with an increased risk of onward HIV transmission^6,7^.

Youth face personal, social, legal and structural barriers to accessing HIV services^8^. Stigma remains much more pronounced for youth because HIV is often associated with taboo behaviors and promiscuity^9^. There remain legal constraints with a requirement for consent from guardians to access HIV services, with a varying age threshold for this requirement that can be as high as 18 years in some countries for independent consent^10,11^. Existing HIV services are mostly verticalized and facility-based and often not geared to address the particular needs of youth. For example, there remains a large unmet need and demand for sexual and reproductive health (SRH) services among youth, including those who are living with HIV^12^, but provision of these alongside HIV prevention and/or care programs remains the exception rather than the rule^9,13^. In addition, judgmental provider attitudes result in poor engagement^9,12^.

Achieving improved HIV outcomes requires that both supply and demand-side barriers be addressed. As HIV services are often not a priority for youth, we hypothesized that integrating the provision of SRH services that are desired and in demand by youth would motivate youth to also take up HIV services. In addition, such a service model, particularly if configured to be youth friendly, could facilitate engagement, be more acceptable and potentially lead to improved program efficiency^14^.

We conducted a cluster-randomized trial (CRT; community-based interventions to improve HIV outcomes in youth—a cluster-randomized trial in Zimbabwe (CHIEDZA)) to investigate the impact of community-based delivery of the whole HIV care cascade (HIV testing, treatment and adherence support) integrated with comprehensive SRH services and general health counseling on population-level HIV outcomes for youth aged 16–24 years. Our rationale was that services situated within communities may be more accessible for youth^15^, and addressing the whole HIV cascade, including HIV testing, linkage to care and support to maintain viral suppression, may minimize the risk of attrition at each step.

## Results

The two-arm CRT was conducted across three provinces in Zimbabwe (Harare, Bulawayo and Mashonaland East), with each province having eight clusters. A cluster was a geographically defined area with a community center from where the CHIEDZA intervention (integrated HIV and SRH services) could be delivered. Thus, a total of 24 clusters were randomized in a 1:1 ratio, stratified by province, to either the control (existing services) or the intervention arm, so that each province had four intervention and four control clusters. Individuals aged between 16 and 24 years and who lived within the boundaries of an intervention cluster were eligible to access the intervention. The deployment of the intervention in the intervention clusters across the provinces was staggered (1 April 2019 to 30 September 2021 in Harare; 1 July 2019 to 15 December 2021 in Bulawayo; 14 October 2019 to 31 March 2022 in Mashonaland East). The uptake of the different CHIEDZA service components, including HIV testing and HIV virological suppression (VS), among CHIEDZA attendees living with HIV is reported separately^16,17^. Briefly, 36,991 youths accessed the CHIEDZA intervention over the 30-month period. Overall, 84.1% of those eligible had at least one HIV test, resulting in 38,603 HIV tests by 29,826 youth, of which 377 were positive (prevalence of newly diagnosed HIV 1.3%). In addition, 1,162 youth accessing CHIEDZA services self-reported being HIV positive^17^.

### Outcome survey participant characteristics

**Fig. 1:**
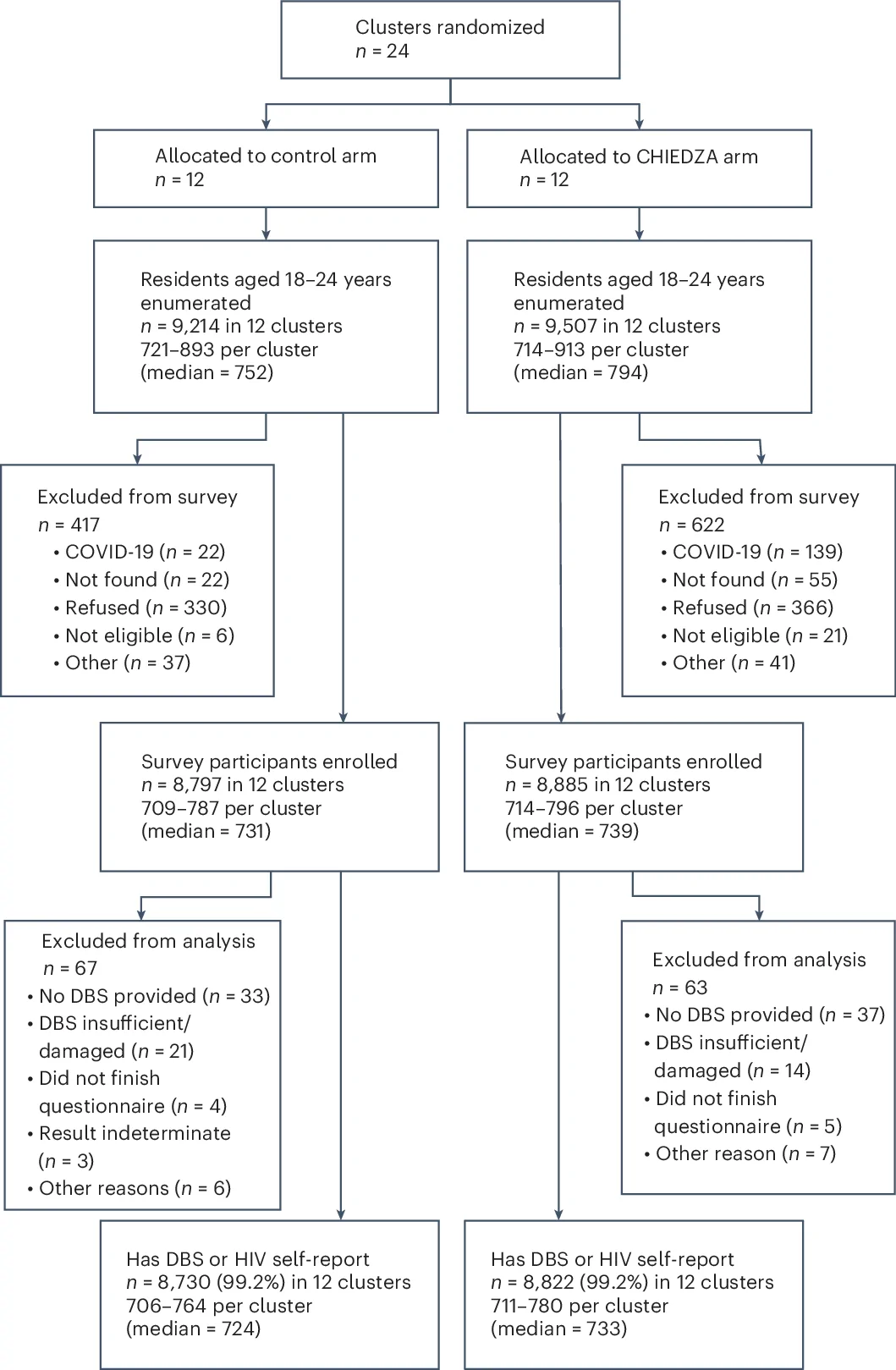
CHIEDZA trial CONSORT diagram. Flowchart of participants in the CHIEDZA endline outcome survey.

The outcome survey was also staggered by province and conducted between 4 October 2021 and 2 June 2022, immediately after the intervention implementation period was completed. In total, 18,721 youth aged 18–24 years resident in households in the randomly selected road segments were enumerated, of whom 17,682 (94.5%) were eligible, gave consent and were enrolled (Fig. 1). Males and older individuals were less likely to be enrolled (Extended Data Table 1). Of those enrolled, 130 (0.7%) were excluded from analysis of the primary outcome due to missing data, leaving 17,552 (Fig. 1).

Overall, 60.8% of participants were female, and the median age was 20 (interquartile range = 19–22) years. The two arms were balanced with respect to sociodemographic characteristics (Table 1).

**Table 1:** Characteristics of outcome survey participants

Parameters	Control arm, *n* (%)	Intervention arm, *n* (%)
	*n* = 8,730	*n* = 8,822
Age (years)		
18–20	4,513 (51.7)	4,660 (52.8)
22–24	4,217 (48.3)	4,162 (47.2)
Gender		
Male	3,539 (40.5)	3,346 (37.9)
Female	5,189 (59.5)	5,476 (62.1)
Non-binary	1 (0.01)	0
Education level attained		
Did not complete primary	168 (1.9)	183 (2.1)
Completed primary	1,489 (17.1)	1,393 (15.8)
Completed form 4	5,337 (61.1)	5,376 (60.9)
Completed form 6	1,036 (11.9)	1,170 (13.3)
Postsecondary	700 (8.0)	700 (7.9)
Main current activity		
In education	2,439 (27.9)	2,482 (28.1)
Formally employed	424 (4.9)	405 (4.6)
Informally employed	1,557 (17.8)	1,588 (18.0)
None of the above	4,310 (49.4)	4,347 (49.3)
Monthly household income		
<US $50	1,452 (19.5)	1,148 (15.2)
US $50–100	2,143 (28.7)	2,281 (30.1)
US $101–200	2,252 (30.2)	2,468 (32.6)
US $201–500	1,312 (17.6)	1,389 (18.3)
>US $500	298 (4.0)	294 (3.9)
Missing	1,273	1,242
Partnership status		
Married or living together	6,595 (75.5)	6,619 (75.0)
Never married	1,705 (19.5)	1,839 (20.9)
Divorced, widowed or separated	430 (4.9)	364 (4.1)
Sexual debut		
Has had penetrative sexual intercourse	5,652 (65.0)	5,554 (63.3)
Never had penetrative sexual intercourse	3,048 (35.0)	3,215 (36.7)
Missing/refused	30	53
Residence at current address		
<12 months	2,051 (23.5)	2,159 (24.5)
12–24 months	770 (8.8)	922 (10.5)
>2 years to 3 years	801 (9.2)	881 (10.0)
>3 years	5,108 (58.5)	4,860 (55.1)

In total, 1,200 participants had a positive HIV test result on their dried blood spot (DBS), and an additional 26 participants were categorized as HIV positive based on their self-report, although their DBS test result was negative (20), indeterminate (4) or missing (2).

A higher proportion in the intervention than control arm reported having ever had an HIV test (71.1% versus 66.1%, *P* = 0.016) and knowing their HIV status (68.5% versus 63.1%, *P* = 0.057). The difference by arm was more pronounced for those who had tested for HIV in the past 12 months (44.4% (intervention) versus 34.7% (control), *P* < 0.001). The HIV prevalence was 6.2% and 7.8% in intervention and control arms, respectively, giving a much larger sample size of youth with HIV (YWH) than the expected 21 per cluster based on an anticipated HIV prevalence of 3%.

**Fig. 2:**
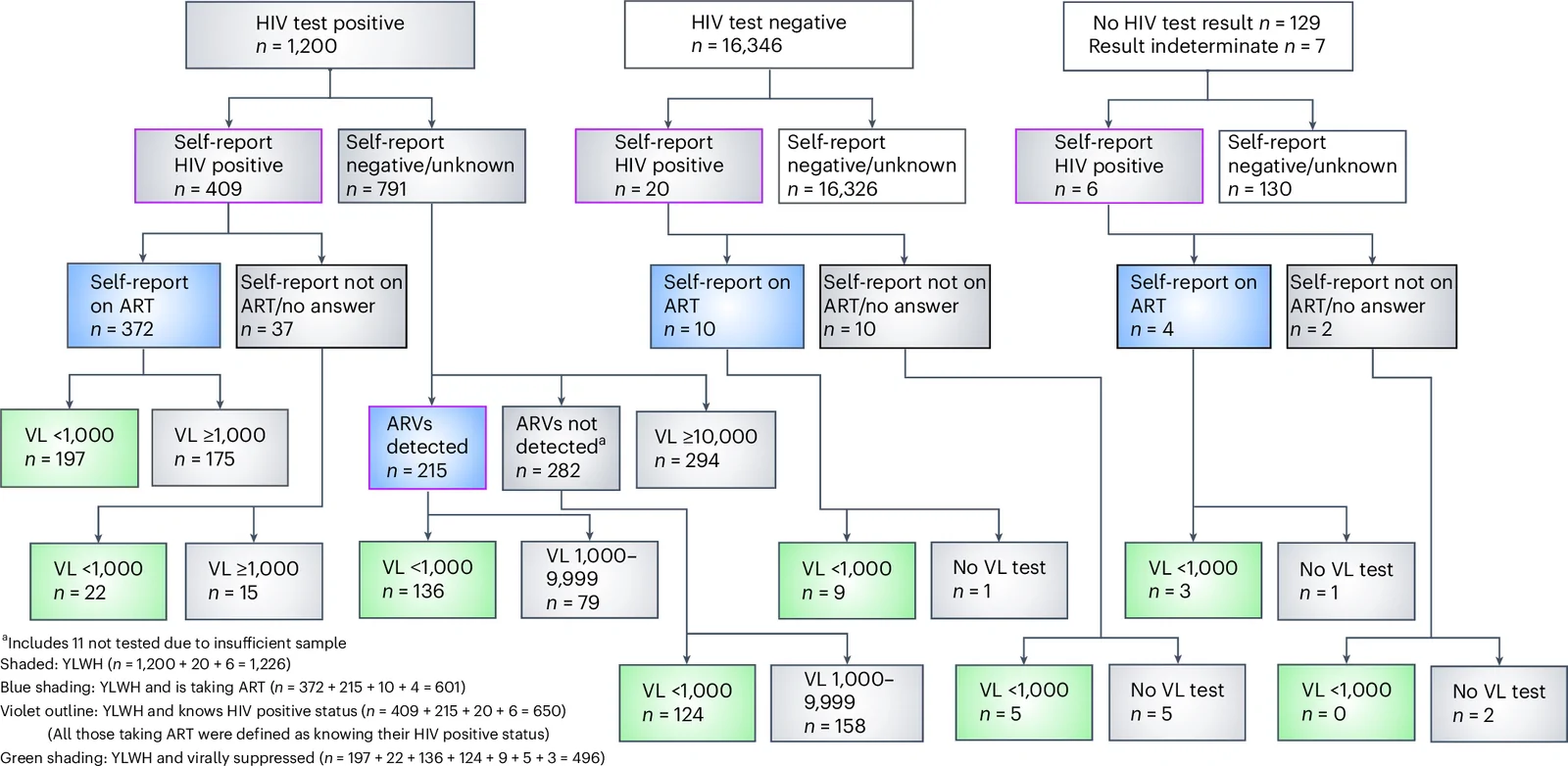
Definition of participants with primary and secondary outcomes. Flowchart showing how participants who had an HIV diagnosis and who were taking antiretroviral therapy were defined. VL, HIV viral load.

Participants were defined as having an HIV diagnosis if they self-reported as HIV positive (*n* = 435) or if ART drugs were detected in their sample (*n* = 211). YWH with an HIV diagnosis were defined as on ART if they self-reported as such (*n* = 386) or if ART drugs were detected *n* = 211; Fig. 2). Notably, of 1,226 YWH overall, 576 (47.0%) were undiagnosed. Of 791 YWH who self-reported as negative or of unknown status, 215 (27.2%) had antiretrovirals (ARVs) detected in their blood sample, while 294 were not tested for ARVs due to high viral load.

### Trial outcomes

#### Primary outcomes

The primary outcome was the proportion of YWH who had VS. There was no difference by arm in the primary outcome (41.3% intervention versus 38.3% control, risk ratio (RR) = 1.07 (95% confidence interval (CI), 0.88–1.30)).

#### Secondary outcomes

The secondary outcomes assessed the effect of the intervention on each step of the HIV care cascade, corresponding to the Joint United Nations Programme on HIV/AIDS (UNAIDS) 90-90-90 targets^18^. There was no difference by arm in the proportion of YWH who had an HIV diagnosis (51.6% versus 51.5%, RR = 0.99 (95% CI, 0.76–1.28); Table 2). In the intervention arm, a lower proportion of diagnosed YWH were taking ART (87.3% (intervention) versus 96.3% (control), RR = 0.91 (95% CI, 0.83–0.99)), but a higher proportion of those taking ART achieved viral suppression (62.7% (intervention) versus 52.6% (control), RR = 1.19 (95% CI, 1.02–1.39)) compared to the control arm. In a sensitivity analysis controlling for factors that were imbalanced between arms among YWH taking ART, namely gender, monthly household income and partnership status (Extended Data Table 2), the RR for achieving viral suppression remained similar (RR = 1.20 (95% CI, 1.08–1.43; *P* = 0.041)).

**Table 2:** Primary and secondary trial outcomes adjusted for gender

Outcome	Sample	*n*	Cluster-level geometric mean prevalence		RR (95% CI)	*P* value	CoV
			Control	Intervention			
Primary outcome							
VS	YWH	1,217	38.3%	41.3%	1.07 (0.88–1.30)	0.47	0.24
Secondary outcomes (aligned to UNAIDS 90-90-90 targets)							
Know the HIV diagnosis	YWH	1,226	51.5%	51.6%	0.99 (0.76–1.28)	0.93	0.25
Taking ART	YWH who know their HIV status	650	96.3%	87.3%	0.91 (0.83–0.99)	0.025	0.08
VS	YWH taking ART	599	52.6%	62.7%	1.19 (1.02–1.39)	0.033	0.06

YWH were defined as participants who either had a positive ELISA test on a DBS sample or self-reported as HIV positive.

YWH who know their HIV status were defined as YWH who either self-reported as HIV positive or had ARVs detected in their DBS sample.

YWH taking ART were defined as YWH who either self-reported as taking ART or had ARVS detected in their DBS sample.

CoV, coefficient of variation.

#### Age- and gender-stratified analyses

When stratified by age and gender, there were no differences by arm in the primary outcome (Table 3). Additionally, there was no statistically significant interaction between study arm and either age or gender for any of the secondary outcomes (Table 3). Three participants had indeterminate HIV test results and did not self-report as HIV positive. In a sensitivity analysis in which they were coded as HIV positive and achieved viral suppression, results were similar to the primary analysis.

**Table 3:** Trial outcomes stratified by age and gender

Outcome			Cluster-level geometric mean prevalence		RR (95% CI)	*P* value	Interaction *P* value
			Control	Intervention			
Primary outcome stratified by gender and age							
VS (in YWH)	Gender	Male	36.9%	42.8%	1.16 (0.79–1.70)	0.43	0.62
		Female	39.7%	41.7%	1.05 (0.87–1.28)	0.60	
	Age (years)	18–20	34.2%	30.2%	0.90 (0.63–1.27)	0.53	0.18
		21–24	39.3%	45.9%	1.16 (0.93–1.44)	0.19	
Secondary outcomes by gender (aligned to UNAIDS 90-90-90 targets)							
Know HIV diagnosis (in YWH)	Male		49.2%	52.3%	1.06 (0.68–1.66)	0.77	0.59
	Female		55.1%	52.7%	0.96 (0.76–1.21)	0.70	
Taking ART (in YWH^a^)	Male		97.6%	88.9%	0.91 (0.83–1.00)	0.062	0.88
	Female		95.6%	87.1%	0.91 (0.83–1.00)	0.046	
VS (in those taking ART)	Male		56.1%	64.1%	1.15 (0.76–1.73)	0.50	0.81
	Female		52.5%	64.5%	1.23 (1.03–1.48)	0.028	
Secondary outcomes by age in years (aligned to UNAIDS 90-90-90 targets)							
Know HIV diagnosis (in YLHW)	18–20		43.0%	50.5%	1.15 (0.64–2.08)	0.62	0.52
	21–24		54.7%	54.1%	0.98 (0.77–1.24)	0.86	
Taking ART (in YLHW^a^)	18–20		95.2%	87.1%	0.92 (0.83–1.02)	0.12	0.92
	21–24		96.2%	87.2%	0.91 (0.82–1.01)	0.06	
VS (in those taking ART)	18–20		52.8%	51.3%	0.98 (0.68–1.43)	0.93	0.11
	21–24		53.7%	68.8%	1.28 (1.11–1.47)	0.002	

^a^Individuals who know their HIV status.

#### Intervention effects among those who accessed the intervention

In a complier average causal effect (CACE) analysis, corresponding to the primary analysis, there was no difference between arms in the primary outcome or in the proportion of YWH who had an HIV diagnosis (Extended Data Table 3). A model of the proportion of diagnosed YWH taking ART failed to converge. Among those taking ART, there was evidence of a higher prevalence of viral suppression in the intervention than in the control arm (RR = 3.85 (95% CI, 1.56, 9.54)). There was little difference between results using the models that used two compliance predictor variables and models that used six (Extended Data Table 3).

#### Effect of distance and length of residence in the cluster on outcomes

Among a subgroup of participants who resided less than the median distance from the community centers from which CHIEDZA services were or would be delivered and who had lived in the clusters for more than 2 years, the RR for the primary outcome in the intervention versus the control arm was higher, but did not reach statistical significance (Extended Data Table 4).

#### Intervention coverage

Based on the estimated population of youth aged 18–24 years in intervention clusters (as estimated from the enumeration in the prevalence survey) and the number of clients who accessed the intervention and would have been the right age for the survey, the intervention coverage would have been approximately 75% of eligible cluster residents (Extended Data Table 5). However, actual reported intervention coverage (proportion of outcome survey participants who reported accessing the intervention in the intervention clusters) was only 23.5% (Extended Data Table 5).

#### Safety and social harms

Two incidents of theft occurred, with study tablets stolen on one occasion and a staff member’s spectacles and a purse on another occasion. These were reported to the police and the community center manager, and security refresher training was conducted. The intervention teams received recurrent threats of closure by security personnel despite clearance for the intervention to continue during the coronavirus disease 2019 (COVID-19) lockdown periods being granted.

There was verbal abuse from some clients on the phone when the intervention staff called clients with HIV to facilitate linkage to care or to attend for follow-up. Subsequent phone follow-ups of these clients were stopped, and these clients were lost to follow-up.

Finally, several clients who had accessed CHIEDZA services were found with condoms at their school and were threatened with suspension. The trial management held a joint discussion with the SRH focal teacher and parents, and suspension was averted.

## Discussion

The starting point for the trial was that existing strategies have not been sufficient to address the disproportionately worse HIV outcomes in youth compared to other age groups. Achieving viral suppression requires an individual to access HIV testing, link to HIV care services and initiate and maintain adherence to ART. The CHIEDZA intervention addressed each of these steps (often termed the HIV care cascade). Delivery of community-based HIV services covering the whole HIV care cascade, including HIV testing, treatment and adherence support, integrated with comprehensive SRH services for youth, had no impact on population-level HIV viral suppression among YWH, the primary outcome. Regarding each step of the HIV care cascade, the intervention did not have an effect on the proportion of YWH who knew their HIV diagnosis. In the intervention arm, a lower proportion of YWH who had an HIV diagnosis were taking ART, but a higher proportion of YWH in the intervention arm taking ART achieved viral suppression than the control arm.

CHIEDZA achieved high levels of HIV testing among those who attended CHIEDZA, with 84% having at least one HIV test^19^. This translated into a population-level impact on HIV testing (ever tested and testing in the past 12 months) and knowledge of HIV status. This is consistent with findings of the Yathu Yathu trial in Zambia, which also offered HIV testing together with SRH services in community hubs to youth and reported substantially increased uptake of HIV testing compared to facility-based testing services, particularly among adolescent boys aged 15–19 years^20^. While HIV testing is a critical first step to accessing HIV treatment, it is also an entry point to HIV prevention services^8^. In addition, engaging with HIV testing once may help overcome anxiety and fear and potentially promote more regular subsequent HIV testing.

However, there was no observed difference by arm in the proportion of YWH who were diagnosed (the first step of the HIV care cascade and one of the secondary outcomes), which likely explains the lack of effect of the intervention on the primary outcome. There are a number of likely reasons for this. First, over the past decade, there has been a scale-up of a range of HIV testing approaches, including the large-scale HIV Self-Testing Africa initiative first launched in Zimbabwe, Malawi and Zambia^21,22^, and this may have diluted any difference between intervention and control clusters.

Second, the effect of the intervention on outcomes was critically dependent not only on the intervention itself but also on coverage, given that outcomes were measured at the population level. We have also shown that both knowledge and usage of CHIEDZA services correlated strongly with distance from community centers^23^. Community sensitization about the intervention was an integral component of the intervention, but this has to be balanced against the increasing risk of contamination. Hence, community mobilizers were used in intervention clusters but could achieve limited geographical coverage within the cluster; media (for example, radio and television advertising) including widely used social media platforms (for example, Facebook and WhatsApp), which could have achieved wider coverage of information about CHIEDZA services, were not used to avoid contamination. Also, intervention coverage may have been compromised by high in- and out-migration among youth. One in four survey participants had been residents in their study community for less than 12 months and therefore had limited exposure to the intervention. We observed that the proportion of participants in the intervention arm who reported accessing the intervention was substantially lower than the estimated coverage calculated as the number of attendees as a proportion of the estimated population of youth in the intervention communities (based on estimates obtained through enumeration). It is therefore possible that while the intervention achieved higher coverage than the 23.5% reported, many individuals who had attended the intervention had subsequently migrated out of the intervention communities. Furthermore, it is possible that individuals who were temporarily or not resident in the clusters, for example, those who resided in neighboring areas, may have accessed the intervention. Indeed, this may explain why estimated coverage was so much higher than reported coverage and even exceeded 100% in some clusters and may have led to underestimation of the intervention’s efficacy. While attendees were asked about their age and address, we relied on attendees’ self-reports to confirm eligibility. Such attendees were not necessarily from the control clusters, which were separated by significant geographical boundaries (such as rivers and major roads) and with sufficient distance between them to avoid contamination^24^. Contamination (that is, individuals resident in control clusters accessing CHIEDZA services) was found to be very low (approximately 3%) when ascertained by comparing the proportion of fingerprint matches of survey participants from the control clusters with those of intervention attendees^23^. Overall, this meant that the participants in the outcome survey were not necessarily representative of individuals who had been exposed to the intervention. In a post hoc analysis among those who had lived longer in the clusters (longer exposure to intervention) and lived closer to the community centers, a stronger effect of the intervention on the primary outcome was observed, although the effect was not statistically significant.

Third, despite the intervention being configured to address the well-recognized demand and supply barriers to HIV testing in youth, it is likely that those at highest risk of being HIV positive were not reached either because they did not access the CHIEDZA services or did not take up HIV testing if they attended. Consistent with Wasserheit and Aral’s theory of phase-specific dynamics of transmission of sexually transmitted infections, as the HIV incidence declines and preventive interventions are established, the sexual and social networks that drive the epidemic become increasingly located within subpopulations that are characterized by higher risk behaviors and less contact with healthcare services^25^.

The proportion of YWH who were on ART (coverage of ART) was high in both trial arms, suggesting that current ART services are performing well for youth who know their HIV positive status and link to care. ART coverage in our population-based survey was higher than that reported in the 2020 population-based impact assessment, which could reflect overall improved access to ART over time^26^. The proportion of YWH who were aware of their HIV status and accessing ART in the intervention arm was lower than that in the control arm. The CHIEDZA intervention provided not just testing but also registration into the ART program, initiation of ART and follow-up care, including adherence support for the duration of the intervention. However, the service was available only once weekly and therefore not at the same frequency as clinic-based services (5–6 days per week). Also, despite counseling, some youth were not yet ready to start ART, which the providers respected^16^. Providers were trained in the provision of youth-friendly services, with autonomy and choice being key aspects. Mobility may also have adversely affected linkage to care. However, this highlights one of many challenges of the provision of HIV treatment to youth^27,28^.

The proportion of youths achieving VS fell far short of the UNAIDS 90-90-90 targets in both arms. The challenges of adherence among young people are well-recognized^29^, and the PHIAs in the region show much lower levels of VS among youth compared to other age groups^3–5^. However, among those YWH taking ART, the proportion who achieved viral suppression was higher in the intervention than in the control arm. This outcome was assessed in individuals who were on ART with the potential for imbalance in characteristics by arm. However, even when adjusting for factors imbalanced between arms, the finding of a higher proportion of YLHW taking ART achieving viral suppression in the intervention arm than the control arm persisted. In a CACE analysis in which outcomes were compared among those who accessed the intervention, the risk of VS among YWH on ART was more than 3.5 times higher in the intervention than in the control arm, suggesting that the intervention may have been effective in supporting adherence to ART. This was likely due to the multimodal approach used to support adherence, including follow-up by providers of those who did not attend for ART refills, specialist counseling and a peer support group. A community-based service model, such as CHIEDZA, could be an effective approach to complement existing HIV treatment services, providing support for adherence and even supporting transition to adult-centered care while providing holistic health services.

While the principal objectives were centered around impact on HIV outcomes, the CHIEDZA trial shows the feasibility of delivering integrated HIV and SRH services to all youth, a group that is often underserved by existing health services. The CHIEDZA service model facilitated the uptake of essential services beyond HIV by youth and addressed an important programmatic gap^17^. Co-designed and co-delivered with youth, it was highly effective in engaging a population that has historically been difficult to reach^30^. Results from an embedded process evaluation (reported separately) showed that the uptake of other services was very high, and the service model was highly acceptable to youth^31^. The model, therefore, provides a practical template for the provision of youth-friendly services. The model complemented, not duplicated, existing facility-based services, was responsive to context and promoted a holistic approach to service delivery, which, while being acceptable to users, may also be programmatically more efficient.

CHIEDZA services were considered ‘critical’ and were selectively endorsed by health authorities to remain open during the COVID-19 pandemic lockdown periods, with operational modifications. An ongoing cost analysis will inform scalability. Such a model offers the potential of incorporating other services such as mental health conditions and substance use, which are not only major causes of morbidity among youth but are also associated with increased risk of HIV infection and worse HIV treatment outcomes^32,33^.

The strengths of the study were the use of an objective biological primary outcome that captured the combined effect across every step of the HIV care cascade, including testing, linkage to and treatment adherence, with secondary outcomes assessing individual steps. The outcome was assessed at the population level and has strong public health relevance, both in terms of impact on individual health outcomes and potentially on HIV transmission, given the overwhelming body of evidence that those who have viral suppression cannot sexually transmit HIV^34^. The study was well-powered, and high participation rates were achieved in the outcome survey. Studies relying solely on self-report may underestimate the proportion of YWH who know their status. It is notable that ARVs were detected in 215 YWH who reported that they were HIV negative or did not know their status, suggesting that they may, in fact, know their HIV positive status and were on treatment. ARV levels were incorporated into our diagnostic algorithm alongside self-report to obtain a more objective measure of knowledge of HIV status.

We acknowledge several limitations. The trial was conducted in urban and peri-urban settings only, as the low population densities in rural areas made a trial of this magnitude unfeasible. Knowledge of HIV status relied partly on self-report, which is subject to social desirability bias and may have resulted in an overestimate of the proportion of undiagnosed HIV among YWH. We did undertake testing of samples for ART levels in those with a viral load of <10,000 copies per ml who did not self-report as being HIV positive. It is, however, possible that a proportion of those with higher viral loads who did not self-report being HIV positive knew their status and were taking ART. There was a 60:40 female-to-male ratio in the outcome survey. While we note that the rate of participation by males was lower than for females, data from the enumeration carried out by the Zimbabwe PHIA in similar areas suggest that this proportion reflects the distribution of females to males in these communities^26^. This was complemented by findings from focus group discussions conducted with study communities, which reported that there are fewer men due to out-migration for employment from urban centers, either to neighboring countries or to agricultural or mining areas^35^. Disseminating information about the CHIEDZA services had to be balanced against the risk of contamination, which may consequently have contributed to lower coverage of the intervention. Implementation of the intervention coincided with the COVID-19 pandemic, which resulted in the shutdown of the intervention for 2 months. This, as well as the subsequent modification of the intervention, likely adversely affected engagement and intervention uptake, particularly among young men^36^. In addition, out-migration may have increased due to the adverse socioeconomic consequences of the pandemic. Also, the CACE analysis uses the latent class method, which depends on assumptions that have not been validated, so the results should be interpreted with some caution.

Integrated HIV and SRH services in community-based settings may overcome some of the demand-side barriers to service provision. Mapping and situating services in non-traditional settings that youth frequent, such as educational institutions and settings where youth socialize, may be needed to improve access. Outreach or mobile services may also help improve coverage. The effectiveness of community-based integrated HIV and SRH services could be improved by wider dissemination of information about services (which was not possible in the context of a trial) and increased frequency of services (for example, daily versus weekly services). This will need to be coupled with ongoing efforts to address community-level stigma and configure services to be youth friendly. Youth-friendly services are unlikely to be achieved with one-off trainings; instead, continual assessment and mentorship will be required.

In summary, there was no effect of the trial intervention on viral suppression among YWH at the population level. However, among those who were diagnosed and accessing ART, the intervention significantly improved viral load suppression. Nearly 50% of YWH remained undiagnosed and were not reached even by services that aimed to address many of the well-known demand and supply barriers to accessing health services. This group needs to be characterized to identify more nuanced strategies for reaching and engaging them, including in HIV care once diagnosed. Innovative and flexible approaches will need to be explored; for example, the use of electronic technologies to provide health-related services and information or ART pick-up points for youth who are mobile.

## Methods

### Study design and setting

CHIEDZA was a parallel open-label two-arm CRT conducted across three provinces in Zimbabwe (Harare, Bulawayo and Mashonaland East). Zimbabwe has experienced an early onset, sustained generalized HIV epidemic with an HIV prevalence of 11.8% in 2020 among adults aged 15–49 years^26^. Harare is the capital and largest city in Zimbabwe, and the population is predominantly of Shona ethnicity; Bulawayo, the second largest city in the country, is situated 440 km from Harare and is predominantly Ndebele. Mashonaland East province borders Harare, and peri-urban settings in this province were selected. In combination, these provinces represented the country’s two main ethnic groups.

A cluster design was used as the intervention was a service that could not be assessed at the level of the individual. A cluster was defined as a geographically demarcated area with an estimated population of approximately 2,000–4,000 youth aged 16–24 years (based on Zimbabwe 2012 Census estimates^37^) that contained a multipurpose community center from which the intervention could be delivered. A cluster had to be serviced by a defined primary care clinic that was not serving another study cluster and was situated within the cluster to ensure integration and collaboration with public-sector services. Where possible, natural boundaries were used to form the edge of the cluster to minimize contamination.

Individuals aged between 16 and 24 years and who lived within the boundaries of an intervention cluster were eligible to access the intervention. Those who were ineligible, that is, self-reported as being outside the eligible age range or living outside the cluster boundaries, were advised to access services at the nearest health facility.

### Randomization and masking

A total of 24 clusters, stratified by province, were randomized in a 1:1 allocation ratio to either the control arm or the intervention arm so that each province had four intervention and four control clusters. A public randomization ceremony was performed in each province, with representatives of the community, the Ministry of Health and Child Care (MoHCC) and respective City Health or town council health departments to ensure transparency and buy-in from stakeholders. Within each province, colored balls were drawn from a bag to allocate each cluster to a trial arm. Given the nature of the intervention, it was not possible to mask either the investigators or the study communities.

### Intervention design and implementation

The intervention was co-designed with relevant stakeholders, including youth and community members (who often serve as gatekeepers to young people accessing services), service providers and policymakers^30^. A key feature of the intervention design process (reported separately) was to center youth—participatory workshops were held with youth to achieve consensus on the intervention’s content and configuration, including the types of services, location of service delivery, types of service providers and the ‘branding’ of the service^30^.

The trial intervention and the logic model showing how the intervention was intended to achieve its intended effects are described in detail in the published trial protocol^24^. In brief, a package of integrated HIV and SRH services was delivered in each intervention cluster. Services included HIV testing—either provider-delivered using a blood-based test or self-testing on site using an oral mucosal test. Those who tested HIV positive or had previously tested HIV positive but were not linked to care were offered a choice of receiving HIV care from the CHIEDZA service, including ART initiation and drug refills, adherence counseling, viral load monitoring and membership of a peer support group or linkage to HIV care at the nearest health facility. Youth who were already in HIV care elsewhere could also opt to receive any of these services from CHIEDZA.

Other services included advice and information on menstrual health and provision of analgesics and reusable menstrual products, pregnancy testing, family planning information, counseling, a choice of short and long-acting contraceptives and emergency contraception, termination of pregnancy, syndromic management of sexually transmitted infections following national guidelines, expedited referral for voluntary male medical circumcision, condoms and HIV risk reduction counseling and general health counseling with onward referral to other health service providers for relevant care where appropriate, for example, mental health issues or intimate partner violence. Information, education and counseling materials about SRH, HIV and general health issues were available in the form of video clips, a health manual available at the centers and online, and a series of short evidence-based SMS messages^38^. All CHIEDZA resources can be found at https://www.chiedza.co.zw/resources.

All services were voluntary (clients could choose whichever services they wanted from a menu card) and free of cost. Tents were pitched within the community center, each of which served as a private consultation area. Confidentiality was a key aspect of the intervention, and, therefore, only age, gender and the service component(s) taken up were recorded for each client who accessed the intervention. Clients were registered using a fingerprint, which was converted into a global unique identification number using SIPMRINTS software (Simprints). Fingerprints were used to record every attendance and track service uptake.

The intervention was configured to be ‘youth friendly’, that is, able to effectively attract youth, meet their needs responsively and retain them in care. Social activities incorporating music, drama, dance, sport and games were held at community centers to increase their engagement with the intervention. A key barrier to youth accessing services is healthcare provider attitudes, and, therefore, ongoing training, supervision and mentorship of providers was an integral component of the intervention, as was community engagement. This included sensitization and peer outreach at locations frequented by youth, including secondary schools. Activities included flyer distribution, information dissemination and in-field live demonstrations of CHIEDZA products (for example, reusable pads, menstrual cups and condoms) to educate, generate support and strengthen community engagement.

Services were provided once weekly on the same day every week in each cluster, except for public holidays, by a multidisciplinary team that included two nurses, four community health workers (CHWs), one counselor and two youth workers. Youth workers provided group education, including product demonstrations and information about menstrual products, registered the clients, organized social activities and were available for informal conversations with clients. CHWs and nurses undertook consultations, and nurses also performed clinical examinations and dispensed ART and contraception. A doctor (a general practitioner with experience in HIV management) was available on the phone to provide advice where required.

The intervention teams underwent a 2-week structured training program. Training covered the following three domains: first, training on clinical aspects such as provision of contraception, management of HIV and sexually transmitted infections and menstrual health management; second, training covered principles of youth-friendliness and communication. This included training on addressing the needs of specific groups, including LGBT+ youth or youth with disabilities; third, training on service delivery and how to operationalize the intervention on a day-to-day basis was provided. Training was guided by a detailed manual of operations and included role plays and hands-on training for certain procedures, for example, using biometrics software and tablets for data capture. Certified training was provided for HIV testing and insertion of implants and intrauterine devices. All staff, including intervention teams, underwent training on good clinical practice. Trial coordinators visited the CHIEDZA services at least once a week to ensure that procedures were being followed, to troubleshoot problems and to provide mentorship to the intervention team members.

Debrief meetings were held every 1–2 months with the intervention teams, whereby challenging consultations and situations were discussed and refresher training was provided. The trial coordinators conducted weekly visits to the CHIEDZA centers to ensure procedures were being followed, to troubleshoot issues and to provide ongoing support and mentorship to the intervention teams.

During the implementation period, the intervention was stopped from April to June 2020 in response to the COVID-19 pandemic. When service delivery restarted, the intervention was modified as follows: face masks and handwashing were mandatory and the number of individuals present at the community center at any time point was restricted, opening hours were shortened and all social activities, group health information sessions and community mobilization activities were stopped. The effects of these adaptations have been reported previously^36,39^.

Originally, a 24-month intervention period was anticipated to achieve optimal coverage within a cluster. An extension of 6 months was added to mitigate against the effects of the COVID-19-related national control measures, including physical distancing, orders to stay home where possible and restrictions on public gatherings. The start date of the intervention was staggered across provinces by 3 months, with Harare province starting first, followed by Bulawayo and then Mashonaland East.

#### Services in the control arm

Other than mapping existing health services (largely facility-based) before trial implementation, the study team delivered no services in the control arm.

### Trial outcomes

Trial outcomes were measured at the population level. The primary outcome was the proportion of YWH who had viral suppression (defined as having an HIV viral load <1,000 copies per ml). The secondary outcomes, reflecting the UNAIDS 90-90-90 targets, were the proportion of YWH who knew their HIV status, the proportion of YWH who knew their HIV status who were taking ART and the proportion of YWH taking ART who had viral suppression, and enabled assessment of the intervention on each step of the HIV care cascade.

YWH were defined as knowing their status if they self-reported as HIV positive or if ARVs were detected. YWH were defined as taking ART if they self-reported as taking it or if ARVs were detected (‘Ascertainment of trial outcomes’). YWH were defined as having viral suppression if their viral load was <1,000 copies per ml, and those who did not have a viral load result were excluded from this outcome.

### Ascertainment of trial outcomes

Outcomes were ascertained through a population-based cross-sectional survey conducted among 18–24-year-olds living in the study clusters at the end of the 30-month intervention period. This age group was chosen to ensure maximal exposure to the intervention. Surveys were conducted in the eight trial clusters in each province over a 3-month period, and the start date of the survey in each province was staggered, reflecting the staggered start date of the intervention^24^.

The sampling methodology for the outcome survey combined remote selection methodologies, incorporating satellite imagery and traditional random street selection. All streets within a cluster were manually split into 100–300 m segments within GIS software (ArcGIS v.10.5), which were then randomly selected. Following community senitization, all households (defined as a person or group of related or unrelated persons who live together in the same dwelling or unit(s) of a dwelling, who acknowledge one male or female as head of the household, who share the same housekeeping arrangements and who are considered a single unit) in each dwelling in the selected street segments were enumerated. All individuals aged 18–24 years residing in the enumerated households were eligible to participate. If a potentially eligible individual was not available at the time of enumeration, up to three repeat visits were made to enroll the individual.

A fingerprint was collected from each participant (as for the intervention). An interviewer-administered questionnaire was used to record sociodemographic data, duration of residence and exposure to the intervention. Participants were asked about knowledge of HIV status, history of HIV testing and care. A DBS sample was collected for anonymized HIV antibody testing and HIV viral load testing (for those who were HIV antibody positive) and for ARV testing (in selected samples).

DBS samples from all YWH who did not self-report as living with HIV and who had a viral load less than 10,000 copies per ml were tested using liquid chromatography tandem mass spectrometry for the presence of ARV drugs—efavirenz, atazanavir, ritonavir, nevirapine, abacavir, lamivudine, zidovudine and dolutegravir. Due to resource constraints, a pragmatic cut-off of 10,000 copies per ml was used to indicate testing for ARV drugs, and higher viral loads were assumed to indicate no treatment being taken. Participants were defined as ‘ARV drugs detected’ if at least one ARV drug was detected, with the exception of lamivudine and efavirenz. If only efavirenz (*n* = 70) or lamivudine (*n* = 4) were detected, the participants were not defined as ‘ARV drugs detected’. Efavirenz has been known to be used as a street drug, and the presence of lamivudine alone could not be explained^40^.

### Sample size considerations

The anticipated sample size was 700 youth per cluster (16,800 total). Assuming a conservative estimate of 3% HIV prevalence among 18–24-year-olds and that the proportion of YWH who had viral suppression was 43% in the control arm (60% diagnosed × 84% on ART × 85% virally suppressed, based on ZIMPHIA estimates), with a coefficient of variation of 0.25, the study would have 80% power to detect a difference of 21% (that is, 64% prevalence of viral suppression in the intervention arm) and 90% power to detect a difference of 24% (67% prevalence of viral suppression). A 66% prevalence of the primary outcome could be achieved by, for example, reaching 80% diagnosis, 91% on ART and 91% viral suppression. With a coefficient of variation of 0.3, the study would have 80% power to detect a difference of 24% and 90% power to detect a difference of 28% in the primary outcome. The estimates of 0.25 and 0.3 for the coefficient of variation were informed by examination of data for urban areas from the ZIMPHIA survey.

The sample size calculation assumed a fixed cluster size of 21 YWH per cluster. The actual cluster size varied between 29 and 77, with a coefficient of variation of 0.3, but this variation would have very little effect on study power.

### Statistical analysis

The statistical analysis plan was finalized before the conduct of the outcome survey (Supplementary Information). Consolidated Standards of Reporting Trials (CONSORT) guidelines for analysis of CRTs were followed with CONSERVE guidelines to report the trial modifications made as a result of the COVID-19 pandemic^41^. Cluster-level analyses were used to adjust for between-cluster variability, as recommended for trials with fewer than 15 clusters per arm. Descriptive analysis was used to compare cluster-level characteristics of the two arms, with adjustment for variables that were unbalanced between arms (avoiding variables likely to be affected by the intervention) and for stratum. The only variable that showed imbalance between arms was gender, and gender was thus adjusted for.

For each outcome, the risk for each cluster was calculated by arm. The mean and s.d. of the log risk were used to estimate the geometric mean and associated 95% CI for each trial arm. A two-stage analysis was conducted using the clan command in Stata 17.0 (ref. ^42^). In the first stage, a logistic regression model was fitted to estimate the effects on the outcome of the adjustment covariates gender and province. Cluster-summarized observed and predicted statistics were used to calculate ratio residuals. In the second stage, linear regression of the log ratio residual on province and arm was used to estimate the RR and 95% CI for the effect of intervention. Significance tests were two-sided with a 5% level of significance. The between-cluster coefficient of variation was calculated as the between-cluster s.d. of the outcome, minus the binomial variation in the outcome within clusters, divided by the mean of the outcome across clusters^43^. Observations with missing values of the outcome were excluded. There were no missing data on adjustment covariates.

#### Prespecified exploratory analyses

Subgroup analysis by gender and age category was conducted to investigate evidence of interaction with the study arm. A sensitivity analysis was conducted in which all indeterminate HIV results were coded as positive and virally suppressed.

The outcomes are affected by coverage (uptake) of the intervention, and, therefore, a CACE analysis was conducted. CACE is an approach used in randomized trials to measure the effect of an intervention on a group of people who would have complied with the intervention they were assigned, to account for non-compliance, which can bias the results of a standard intent-to-treat analysis^44^. Compliance was defined as attending the CHIEDZA service, and CACE was used to estimate the effect of the trial among participants who attended the CHIEDZA services, by comparing intervention arm participants who attended the CHIEDZA service with comparable individuals in the control arm^45^. Survey participants in the intervention arm were coded as compliers if they either had a fingerprint match to CHIEDZA service clients or self-reported attending the CHIEDZA service. Structural equation modeling was used to create two latent classes, with all compliers in the intervention arm in one class and all non-compliers in the other. The control arm participants were allocated to the two classes based on their similarity to the intervention arm compliers and non-compliers, on the matching variables used in the model. Within the ‘complier’ class, a generalized linear model with a binomial family and logit link was run to estimate the effect of the intervention in this group. The latent class modeling was run twice—first, with two predictor variables for class (length of residence and living within median proximity to the community center), and second, with six variables predictive of compliance (gender, living within median proximity to the community center, length of residence, sexual debut, age as a binary variable and socioeconomic status quintile).

#### Post hoc analyses

The effect of distance from residence to the CHIEDZA intervention centers and the length of residence in the cluster on the primary outcome was investigated. Intervention uptake as reported in the survey sample was compared to intervention coverage based on estimates of the population of youth residents in the cluster to explore possible effects of population turnover on outcomes. We used the enumeration data from the survey to estimate the total population of 18–24-year-olds in each cluster. The number of 18–24-year-olds enumerated was divided by the proportion of the cluster area that was surveyed. We used the date of birth of CHIEDZA service attendees to determine the number of clients in each cluster who were aged 18–24 years when the survey began. The number of clients per cluster divided by the estimated number of youth residents in the cluster indicated service coverage, assuming all clients were still residing in the cluster at the time of the survey.

### Safety and adverse events

Given that the outcome was ascertained through an endline survey, no midpoint evaluation was conducted. A data monitoring committee was not established, as no major safety concerns were expected. An active incident recording and management system was in place to address any untoward events that risked or resulted in actual harm to participants and staff. This was discussed with the trial principal investigator, and appropriate steps were taken.

### Protocol deviations and amendments

Any protocol deviation was to be reported to the ethics committees and the Trial Steering Committee. When political events were being hosted at the multipurpose community centers, intervention providers were denied entry. When this occurred, tents were pitched, and these served as consultation booths. During the COVID-19 pandemic, there were adaptations to intervention service delivery, including reduced working hours, use of personal protective equipment and a halt to all social activities, and the impacts of these have been reported^36^.

All protocol changes were determined based on consultation with the Trial Steering Committee and the research team and were approved by all ethics committees.

### Inclusion and ethics statement

The research was undertaken as part of a longstanding partnership between the Biomedical Research and Training Institute (BRTI), Zimbabwe and the London School of Hygiene and Tropical Medicine (LSHTM), which has led to many collaborative studies investigating HIV epidemiology in adolescents and young people and interventional studies to improve outcomes across the HIV care cascade. The principal investigator and several LSHTM co-investigators have been based in Zimbabwe for many years. Discussions with the MoHCC and previous work from this region were used to guide the design of this study and have been taken into account in the citations for this paper.

In keeping our focus on developing contextually relevant interventions, Zimbabwean study team members led the intervention design, selection of the study locations, conduct of randomization ceremonies, implementation and evaluation of the intervention and community engagement activities. Specifically, formative research was undertaken with guardians, community members and youth, and participatory co-design workshops were held with youth to maximize relevance and acceptability. The trial was coordinated by Zimbabwean researchers, and all team members collaborated on data ownership, intellectual property and authorship of publications related to the work.

Three members of the Zimbabwean research team embedded their doctoral research within the CHIEDZA trial. In addition, 3 master’s and 3 bachelor’s degrees and 11 diploma courses for Zimbabwean staff were supported within the CHIEDZA project.

Providers were specifically trained on how to communicate with and address the specific needs of LGBT+ clients. Where feasible, the needs of people with disabilities were addressed, for example, by identifying communicators who were able to use sign language. Standard operating procedures and training ensured that providers were safe, for example, postexposure prophylaxis, safe lifting and handling procedures and keeping safe after hours. During the COVID-19 pandemic, the intervention was considered a critical service during COVID-19 lockdowns and was allowed to continue. The intervention was, however, reconfigured to ensure that both staff and clients were safe. Personal protective equipment was provided, and staff were trained on infection prevention and control procedures.

### Ethics approval

Ethical approval was granted by the Medical Research Council of Zimbabwe (reference MRCZ/A/2387), the Institutional Review Board of the BRTI (reference AP149/2018) and the LSHTM Research Ethics Committee (reference 12063).

Zimbabwe national guidelines stipulate that those aged 16 years and older can give independent consent to accessing HIV and SRH services. At the level of the intervention, as each of the individual service components was established public health intervention (for example, HIV testing, HIV care and family planning), consent was implied when clients took up intervention activities and specific written consent to participate was not obtained.

For the outcome survey, written consent was obtained from participants. To facilitate age-appropriate and informed consent, eligible individuals were shown a video of the study procedures enacted and narrated by the study team on a tablet with narration in English, Shona or Ndebele. The video was also available online for participants to watch later and participants were given a brief and simple information sheet to keep. Consent was documented electronically through a signature or fingerprint on the tablet, with a signed paper copy retained by participants.

### Reporting standards

The trial is registered at ClinicalTrials.gov registration: NCT03719521. The intervention is described in accordance with the template for intervention description and replication checklist (Supplementary Information). The trial is reported in accordance with CONSORT for CRTs (Supplementary Information)^46^.

### Reporting summary

Further information on research design is available in the Nature Portfolio Reporting Summary linked to this article.

## Online content

Any methods, additional references, Nature Portfolio reporting summaries, source data, extended data, supplementary information, acknowledgements, peer review information; details of author contributions and competing interests; and statements of data and code availability are available at https://doi.org/10.1038/s41591-025-03762-z.

## Supplementary information

Reporting Summary

## Figures and Tables

**Extended Data Table 1:**
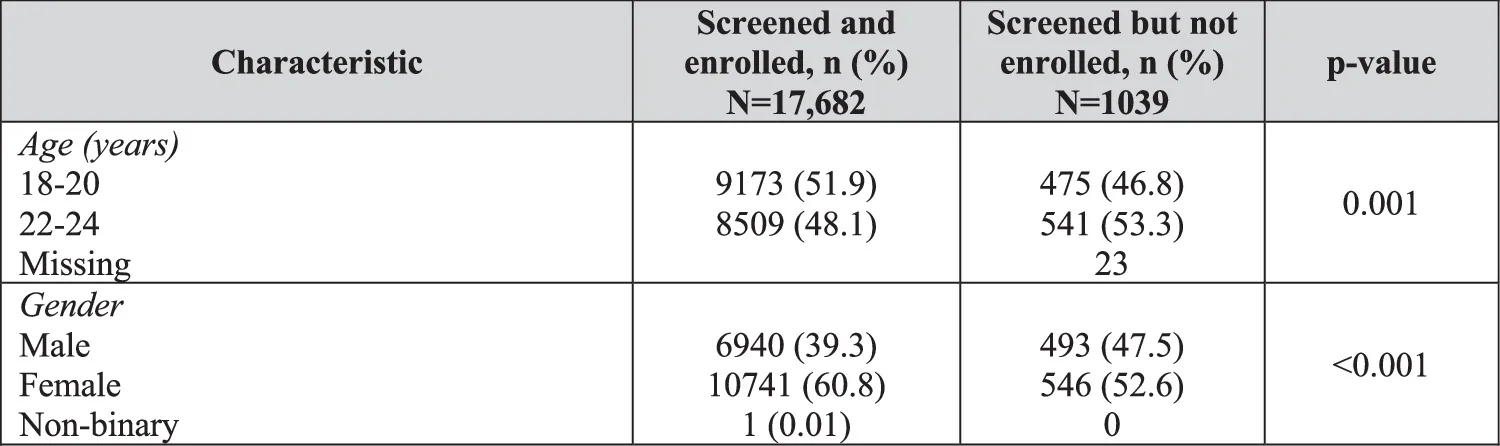
Characteristics of enrolled and not enrolled participants

**Extended Data Table 2:**
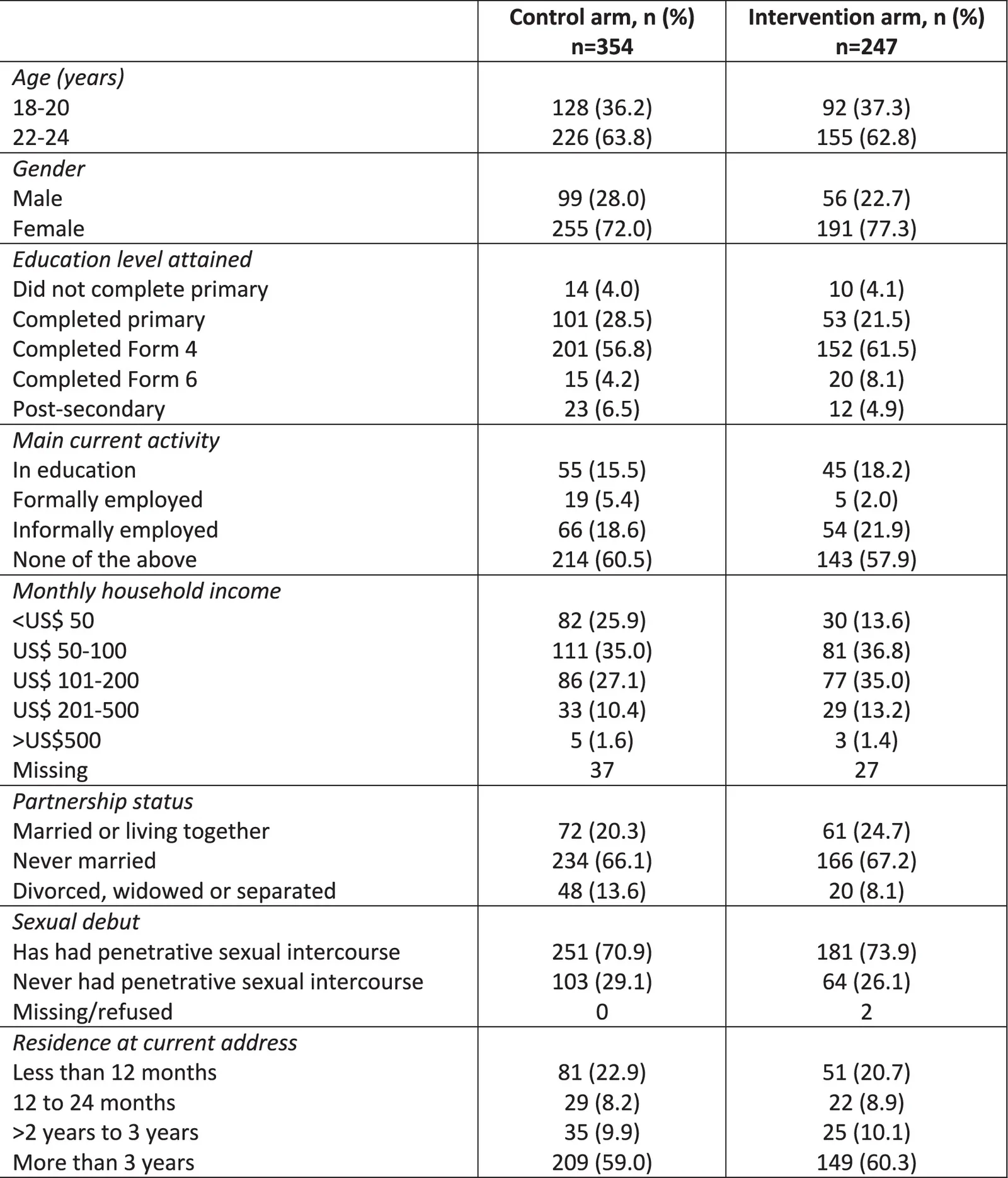
Characteristics of participants taking ART by trial arm

**Extended Data Table 3:**
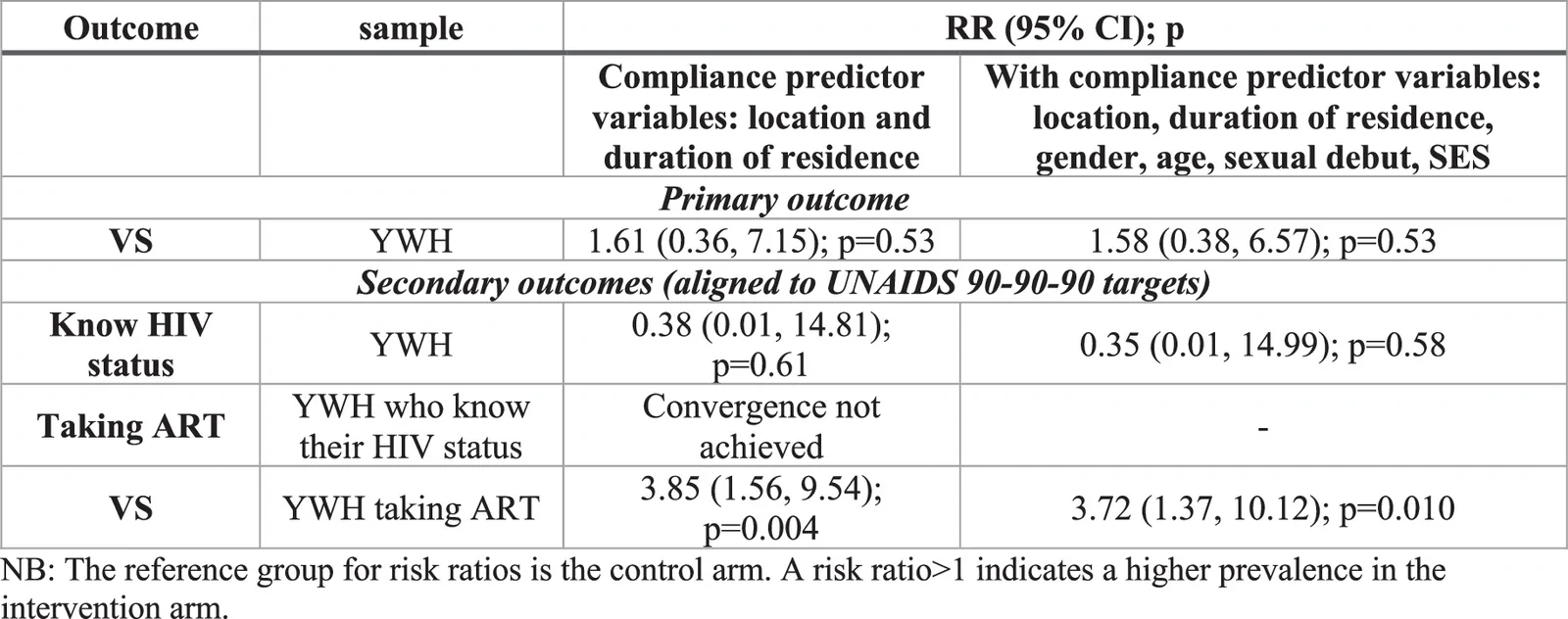
CACE analysis trial effects

**Extended Data Table 4:**
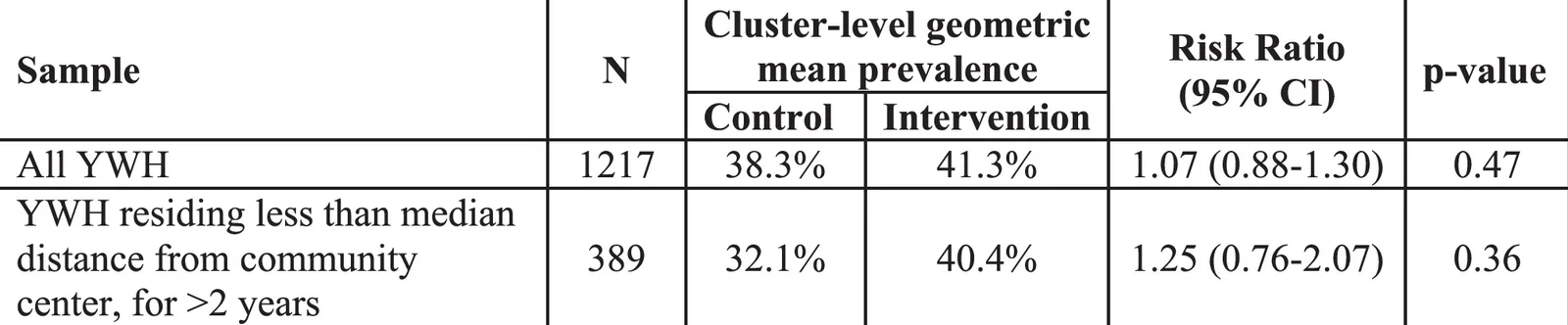
Primary outcome by distance from cluster and length of residence

**Extended Data Table 5:**
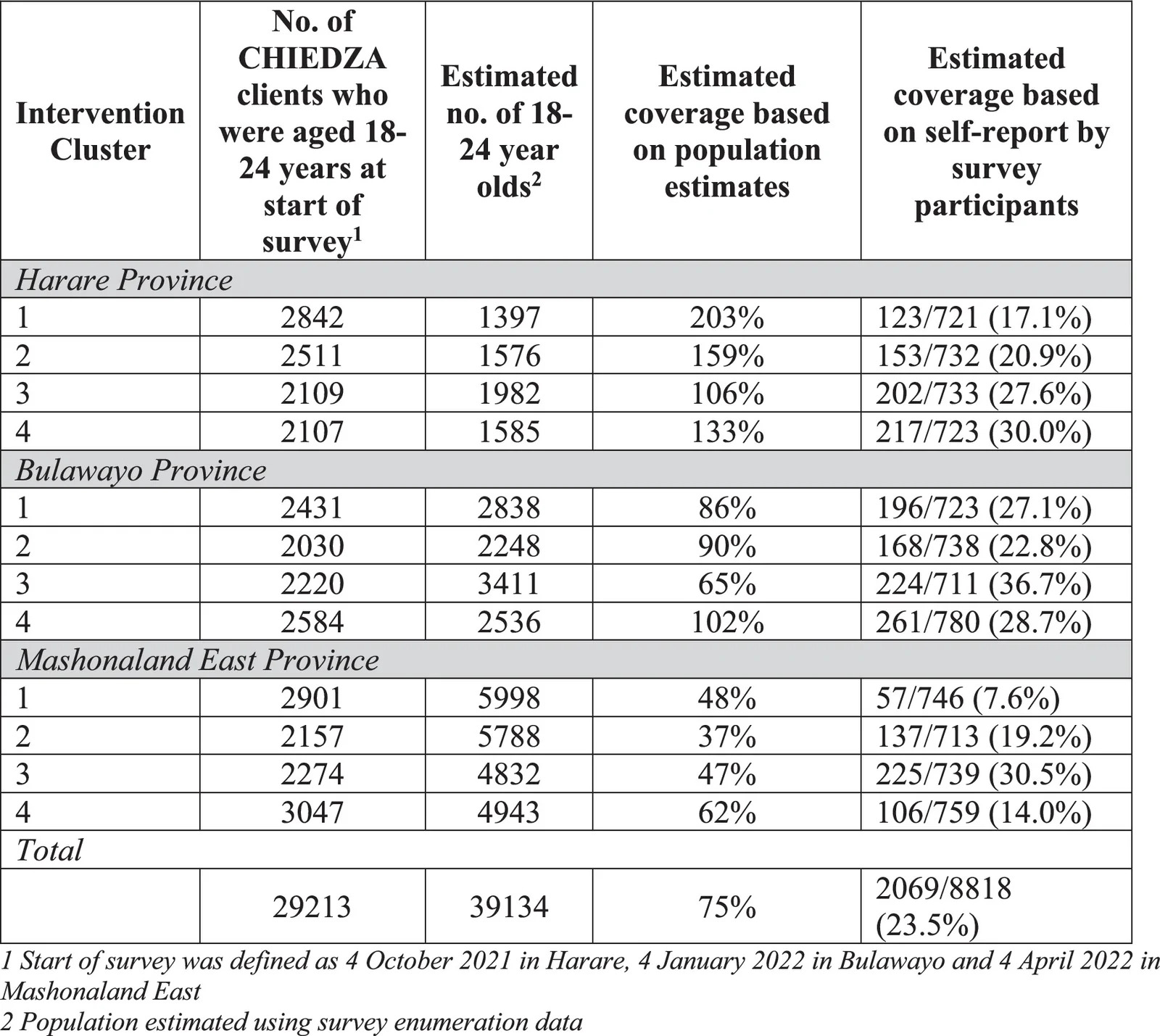
Estimated and actual coverage of the intervention by cluster

## Data Availability

Requests for data should be sent to the corresponding author, R.A.F. Requests will be considered by the CHIEDZA Trial Management Group, which includes the principal investigator, data manager, statistician and the trial coordinator. Responses to requests for data will be provided within 2 weeks and will be communicated by the corresponding author. Data analyzed in this paper were collected with an ethical commitment that they would be accessed by authorized users and used for study purposes only. Limited access to a data subset is permitted for research auditing and validation, subject to the signing of a licence agreement. A request form can be completed at https://doi.org/10.17037/DATA.00004651.

## Code availability

Processing code and documentation, including the study protocol and the manual of operations, have been made openly available under a Creative Commons licence at https://doi.org/10.17037/DATA.00004651.
