# Author Correction: Integrated community-based HIV and sexual and reproductive health services for youth: a cluster-randomized trial

**DOI:** 10.1038/s41591-026-04581-6

**Published:** 2026-07-16

**Authors:** Rashida A. Ferrand, Ethel Dauya, Chido Dziva Chikwari, Tsitsi Bandason, Sarah Bernays, Constance Mackworth-Young, Aoife M. Doyle, Chris Grundy, Pitchaya Indravudh, Fern Terris-Prestholt, Constancia Vimbayi Mavodza, Owen Mugurungi, Tsitsi Apollo, Getrude Ncube, Leyla Larsson, Ona McCarthy, Victoria Simms, Mandikudza Tembo, Katharina Kranzer, Richard J. Hayes

**Affiliations:** 1 Clinical Research Department, London School of Hygiene & Tropical Medicine, London, UK; 2 The Health Research Unit Zimbabwe, Biomedical Research and Training Institute, Harare, Zimbabwe; 3 Department of Infectious Disease Epidemiology, London School of Hygiene & Tropical Medicine, London, UK; 4 Faculty of Medicine and Health, University of Sydney, Sydney, New South Wales Australia; 5 Department of Global Health and Development, London School of Hygiene & Tropical Medicine, London, UK; 6 Department of Public Health, Environments and Society, London School of Hygiene & Tropical Medicine, London, UK; 7 AIDS and TB Unit, Ministry of Health and Child Care, Harare, Zimbabwe; 8 Institute of Infectious Diseases and Tropical Medicine, LMU University Hospital, Munich, Germany; 9 Department of Population Health, London School of Hygiene & Tropical Medicine, London, UK

**Keywords:** Epidemiology, Developing world

Correction to: *Nature Medicine* https://doi.org/10.1038/s41591-025-03762-z, published online 24 June 2025.

In the version of the article initially published, Fern Terris-Prestholt’s name appeared incorrectly (as Terris-Presholt) and has now been corrected in the HTML and PDF versions of the article.

