# Supplementary material for: Integrated community-based HIV and sexual and reproductive health services for youth: a cluster-randomized trial
Source: Nat Med. 2025 Jun 24;31(9):3081–8. doi: 10.1038/s41591-025-03762-z (PMC7617871; doi:10.1038/s41591-025-03762-z)
Supplement: Supplementary file 1 — Reporting Summary [file 41591_2025_3762_MOESM1_ESM.pdf]

nature portfolio

Corresponding author(s): Rashida Ferrand

Last updated by author(s): May 13, 2025

Reporting Summary

Nature Portfolio wishes to improve the reproducibility of the work that we publish. This form provides structure for consistency and transparency in reporting. For further information on Nature Portfolio policies, see our Editorial Policies and the Editorial Policy Checklist.

Statistics

For all statistical analyses, confirm that the following items are present in the figure legend, table legend, main text, or Methods section.

n/a	Confirmed	
☐	☒	The exact sample size (*n*) for each experimental group/condition, given as a discrete number and unit of measurement
☒	☐	A statement on whether measurements were taken from distinct samples or whether the same sample was measured repeatedly
☐	☒	The statistical test(s) used AND whether they are one- or two-sided<br>*Only common tests should be described solely by name; describe more complex techniques in the Methods section.*
☐	☒	A description of all covariates tested
☐	☒	A description of any assumptions or corrections, such as tests of normality and adjustment for multiple comparisons
☐	☒	A full description of the statistical parameters including central tendency (e.g. means) or other basic estimates (e.g. regression coefficient) AND variation (e.g. standard deviation) or associated estimates of uncertainty (e.g. confidence intervals)
☐	☒	For null hypothesis testing, the test statistic (e.g. *F*, *t*, *r*) with confidence intervals, effect sizes, degrees of freedom and *P* value noted<br>*Give P values as exact values whenever suitable.*
☒	☐	For Bayesian analysis, information on the choice of priors and Markov chain Monte Carlo settings
☒	☐	For hierarchical and complex designs, identification of the appropriate level for tests and full reporting of outcomes
☒	☐	Estimates of effect sizes (e.g. Cohen's *d*, Pearson's *r*), indicating how they were calculated

Our web collection on statistics for biologists contains articles on many of the points above.

Software and code

Policy information about availability of computer code

Data collection	Clients were registered using a fingerprint which was converted into a Global Unique Identification number using SIPMRINTS software (SIMPRINTS, Cambridge, UK). Data was collected on electronic tablets using SurveyCTO Collect v2.81 software
Data analysis	Data was analyzed using STATA (version 17.0)

For manuscripts utilizing custom algorithms or software that are central to the research but not yet described in published literature, software must be made available to editors and reviewers. We strongly encourage code deposition in a community repository (e.g. GitHub). See the Nature Portfolio guidelines for submitting code & software for further information.

Data

Policy information about availability of data

All manuscripts must include a data availability statement. This statement should provide the following information, where applicable:

- Accession codes, unique identifiers, or web links for publicly available datasets
- A description of any restrictions on data availability
- For clinical datasets or third party data, please ensure that the statement adheres to our policy

Requests for data should be sent to the corresponding author. Responses to requests for data will be provided within two weeks and will be communicated by the corresponding author. Data analysed in this paper was collected with an ethical commitment that it would be accessed by authorised users and used for study purposes only. Limited access to a data subset is permitted for research auditing and validation, subject to the signing of a licence agreement. A request form can be

completed at https://doi.org/10.17037/DATA.00004651. Processing code and documentation including the study protocol and the Manual of Operations have been made openly available under a Creative Commons licence at https://doi.org/10.17037/DATA.00004651.

Research involving human participants, their data, or biological material

Policy information about studies with human participants or human data. See also policy information about sex, gender (identity/presentation), and sexual orientation and race, ethnicity and racism.

Reporting on sex and gender	Yes, Described in Manuscript
Reporting on race, ethnicity, or other socially relevant groupings	Described in Manuscript
Population characteristics	Overall, 60.8% of participants were female and the median age was 20 (IQR 19-22) years. The two arms were balanced with respect to socio-demographic characteristics.
Recruitment	Individuals aged 16–24 years who lived within the boundaries of an intervention cluster were eligible to access intervention. Trial outcomes were ascertained through a population-based cross-sectional survey in study clusters. Sampling methodology used satellite imagery to select random 100—300 meter street segments using ArcGIS (v10.5). Households in selected segments were enumerated. All individuals aged 18–24 years residing in the enumerated households were eligible to participate.
Ethics oversight	Ethical approval was granted by the Medical Research Council of Zimbabwe (MRCZ/A/2387), the Institutional Review Board of the Biomedical Research and Training Institute (AP149/2018) and the London School of Hygiene & Tropical Medicine (LSHTM) Research Ethics Committee (12063).

Note that full information on the approval of the study protocol must also be provided in the manuscript.

Field-specific reporting

Please select the one below that is the best fit for your research. If you are not sure, read the appropriate sections before making your selection.

☒ Life sciences ☐ Behavioural & social sciences ☐ Ecological, evolutionary & environmental sciences

For a reference copy of the document with all sections, see nature.com/documents/nr-reporting-summary-flat.pdf

Life sciences study design

All studies must disclose on these points even when the disclosure is negative.

Sample size	Assuming estimate of 3% HIV prevalence among 18–24-year-olds and that the proportion of YWH who had viral suppression was 43% in the control arm (60% diagnosed × 84% on ART × 85% virally suppressed, based on ZIMPHIA estimates), with a coefficient of variation of 0.25, the study would have 80% power to detect a difference of 21% (i.e. 64% prevalence of viral suppression in the intervention arm) and 90% power to detect a difference of 24% (67% prevalence of viral suppression). The estimates of 0.25 and 0.3 for the coefficient of variation were informed by ZIMPHIA data.
Data exclusions	Of 17682 enrolled, 130 (0.7%) excluded from analysis of primary outcome due to missing data leaving 17552
Replication	Not feasible to repeat an outcome survey
Randomization	24 clusters, stratified by province, were randomized 1:1 allocation ratio to either the control arm or the intervention arm, so that each province had four intervention<br>and four control clusters. A public randomization ceremony was performed in each province, to ensure transparency and buy-in from stakeholders.
Blinding	Given the nature of the intervention (services), it was not possible to mask either investigators or study communities.

Reporting for specific materials, systems and methods

We require information from authors about some types of materials, experimental systems and methods used in many studies. Here, indicate whether each material, system or method listed is relevant to your study. If you are not sure if a list item applies to your research, read the appropriate section before selecting a response.

Materials & experimental systems

n/a	Involved in the study
☒	☐ Antibodies
☒	☐ Eukaryotic cell lines
☒	☐ Palaeontology and archaeology
☒	☐ Animals and other organisms
☐	☒ Clinical data
☒	☐ Dual use research of concern
☒	☐ Plants

Methods

n/a	Involved in the study
☒	☐ ChIP-seq
☒	☐ Flow cytometry
☒	☐ MRI-based neuroimaging

Clinical data

Policy information about clinical studies

All manuscripts should comply with the ICMJE guidelines for publication of clinical research and a completed CONSORT checklist must be included with all submissions.

Clinical trial registration: Trial is registered with Clinical Trials.gov (Trial registration number: NCT03719521)

Study protocol: Published and cited in manuscript

Data collection: Trial conducted across three provinces in Zimbabwe (Harare, Bulawayo, and Mashonaland East), with 8 clusters/province. Deployment of intervention across provinces was staggered (1st April 2019-30th September 2021 in Harare; 1st July 2019-15th December 2021 in Bulawayo; 14th October 2019-31st March 2022 in Mashonaland East. Outcome survey was staggered by province and conducted between 4th October 2021- 2nd June 2022

Outcomes: Trial outcomes measured at population level. Primary outcome: proportion of youth with HIV (YWH) who had viral suppression (HIV viral load <1000 copies/ml). Secondary outcomes, reflecting the UNAIDS 90-90-90 targets, were proportion of YWH who knew their HIV status, proportion of YWH who knew their HIV status who were taking ART, and proportion of YWH taking ART who had viral suppression.

Plants

Seed stocks: N/A

Novel plant genotypes: N/A

Authentication: N/A
